# A Flexible Wireless Sensor Network Based on Ultra-Wide Band Technology for Ground Instability Monitoring

**DOI:** 10.3390/s18092948

**Published:** 2018-09-05

**Authors:** Lorenzo Mucchi, Sara Jayousi, Alessio Martinelli, Stefano Caputo, Emanuele Intrieri, Giovanni Gigli, Teresa Gracchi, Francesco Mugnai, Massimiliano Favalli, Alessandro Fornaciai, Luca Nannipieri

**Affiliations:** 1 Department of Information Engineering, University of Florence, via di S. Marta 3, 50139 Florence, Italy; sara.jayousi@unifi.it (S.J.); alessio.martinelli@unifi.it (A.M.); stefano.caputo@unifi.it (S.C.); 2 Department of Earth Sciences, University of Florence, via La Pira 4, 50121 Florence, Italy; emanuele.intrieri@unifi.it (E.I.); giovanni.gigli@unifi.it (G.G.); teresa.gracchi@unifi.it (T.G.); mugnai.francesco@gmail.com (F.M.); 3 National Institute of Geophysics and Volcanology (INGV), via della Faggiola 32, 56126 Pisa, Italy; massimiliano.favalli@ingv.it (M.F.); alessandro.fornaciai@ingv.it (A.F.); luca.nannipieri@ingv.it (L.N.)

**Keywords:** Ultra-Wide Band, wireless sensor networks, monitoring, warning system, ground instability, landslide, time of flight, two-way ranging

## Abstract

An innovative wireless sensor network (WSN) based on Ultra-Wide Band (UWB) technology for 3D accurate superficial monitoring of ground deformations, as landslides and subsidence, is proposed. The system has been designed and developed as part of an European Life+ project, called Wi-GIM (Wireless Sensor Network for Ground Instability Monitoring). The details of the architecture, the localization via wireless technology and data processing protocols are described. The flexibility and accuracy achieved by the UWB two-way ranging technique is analysed and compared with the traditional systems, such as robotic total stations (RTSs) and Ground-based Interferometric Synthetic Aperture Radar (GB-InSAR), highlighting the pros and cons of the UWB solution to detect the surface movements. An extensive field trial campaign allows the validation of the system and the analysis of its sensitivity to different factors (e.g., sensor nodes inter-visibility, effects of the temperature, etc.). The Wi-GIM system represents a promising solution for landslide monitoring and it can be adopted in combination with traditional systems or as an alternative in areas where the available resources are inadequate. The versatility, easy/fast deployment and cost-effectiveness, together with good accuracy, make the Wi-GIM system a possible solution for municipalities that cannot afford expensive/complex systems to monitor potential landslides in their territory.

## 1. Introduction

### 1.1. Background and Motivation

Continuous and reliable field monitoring, possibly associated with early warning systems, is essential for hazard assessment and ground instability risk management. Different monitoring techniques are used to measure the relevant parameters, such as ground displacements, ground and surface water conditions and climatic parameters.

A large number of different techniques for displacement monitoring has been made available to geoscientists in recent years [1,2]. The monitoring instruments typically include: extensometers, borehole inclinometers, superficial tilt-meters and clinometers, topographic instruments, satellite interferometry, Ground-based interferometric Synthetic Aperture Radar (GB-InSAR) [3,4,5,6] and robotic total stations (RTSs) [7,8,9,10].

Moreover, Global Navigation Satellite System (GNSS) has been proposed to overcome the need of line of sight (LOS) condition and to provide high-precision 3D monitoring [11,12,13,14]. The adoption of this technology to landslide monitoring is driven by the the development of low-cost GNSS equipment.

Data storage and transmission are still in most cases based on cable connections between sensors, data loggers and General Packet Radio Service (GPRS) modems. The need of high maintenance and the presence of a single point of failure make wiring each sensor to a central data logger infeasible due to the increase of installation and maintenance cost and time.

Recently, in the case of a catastrophic event, wireless sensor network (WSN) technology is often used to support environmental detection, monitoring and prediction, thanks to its capability of real-time monitoring of natural phenomena and data extraction. Some of the main advantages of the adoption of WSN for environmental real-time monitoring are: fast and easy large scale deployment, low maintenance, scalability and adaptability to different scenarios. On the other hand, some drawbacks are represented by memory, power and throughput. Focusing on the use of WSNs for landslide monitoring [15], several parameters, such as temperature and humidity, are gathered by multiple sensors and sent to a control centre; however, sensors are not directly used to measure the ground movement. New methodologies for ground instability measurement based on distributed and connected low cost wireless sensors have been proposed and are becoming more and more widespread.

A distributed strain sensors system for landslide prediction is presented [16], while a WSN capable of measuring the strain in the rock due to the build up pressure is proposed in [17]. Benefits and limits of the WSN and the methods implemented and deployed in the northern Italy Apennines for landslide monitoring are described in [18]. Slope movement are detected thanks to an accelerometer included in each node, as well as temperature, pressure and humidity, which are monitored by additional integrated instruments. In [19] the ground motions are measured by surface mining through three-axial accelerometer units.

These experiences highlight important advantages of the WSN over traditional ground-based monitoring techniques, such as: (i) capability of collecting, aggregating and analyzing, from a multi-point perspective, diverse and distributed data; (ii) possibility to be very quickly deployed without requiring any pre-existing infrastructure; (iii) limited-cost wide area coverage, thanks to the exploitation of multi-hop communications; (iv) existence of low-cost energy-efficient algorithms to allow the network to run for months without human intervention; (v) reduced vulnerability and environmental impact due to the adoption of wireless communications with respect to physical wiring among sensors and data loggers; and (vi) possibility of coexistence and integration with existing instruments, acting as an infrastructure to collect, process, and transmit data to a remote control center. It is important to note that the WSNs have been mainly used to move information, not to measure the movements with a radio frequency technology. The measure of the ground movement is made by inertial sensors, or classical extensometers, or other traditional device, which is then interconnected by the WSN to a data logger.

The diffusion of WSNs for landslide monitoring has been limited by four main factors: (i) accuracy of the measure; (ii) energy consumption; (iii) need of post-processing of acquired data; and (iv) capability of monitoring only a few points.

### 1.2. Objectives and Project Framework

To overcome these limits, an innovative WSN based on the adoption of ultra wideband (UWB) technology for the 3D accurate superficial monitoring of ground deformations, such as landslides and subsidence, is proposed here. The main idea is to detect the surface movements by acquiring the position of many sensor nodes (organized in clusters) distributed over the monitored area. Each sensor node uses the UWB two-way ranging technique to measure the distance with all the others. The main features of the proposed system are: (i) easy and quick installation, (ii) flexibility and capability to adapt to different application scenarios, (iii) cost effectiveness, and (iv) accuracy, also in non light of sight (NLOS) conditions.

These activities have been carried out in the framework of the Wi-GIM project (Wireless sensor network for Ground Instability Monitoring) within the European Life+ financing program for the environment [20]. Addressing the increase of landslide phenomena, which cause damage to buildings, infrastructures, territory, and population, the solution is the monitoring of phenomena and the (early) warning of the resident population in risky areas.

The Wi-GIM project aims to obtain a geological monitoring system that reduces the installation time with respect to the more traditional instruments, and consequently reduces the risks for operational personnel involved in the field activities, as well as promptly warns the civilians in areas at risk. An innovative landslides monitoring system (displacement, subsidence) capable of providing high precision, rapid installation, optimization of energy management and cost-effective investments has been designed, developed and tested through an extensive experimental campaign.

## 2. Materials and Methods

The objective of this section is to describe the proposed solution for ground deformation monitoring. In particular, the Wi-GIM architecture, able to perform accurate measurements of the ground movements, is presented, highlighting the adopted localization technology, the communication protocols, the management algorithms and the post-processing methods. The methodologies followed to design and develop the system are also reported.

### 2.1. Wi-GIM System Architecture

The WI-GIM system is designed to yield accurate measurements of ground movements by defining a *grid* over the soil surface to be used to monitor landslide movements in an efficient and cost-effective way, allowing an easy and quick deployment over a risk area of a large amount of sensors.

**Figure 1:**
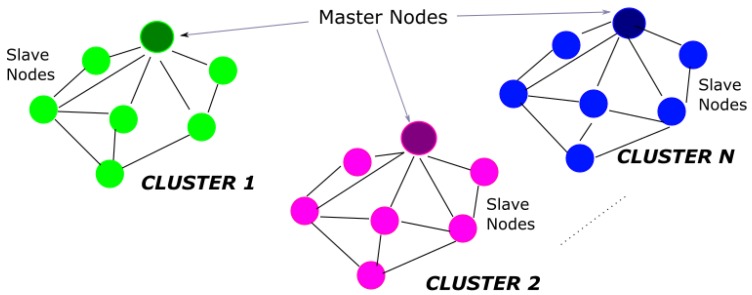
Wi-GIM network topology.

As depicted in Figure 1, an ad hoc wireless sensor network based on a modified star topology is designed. The master and slave paradigm, together with clustering technique, is adopted to allow a versatile and energy-aware estimation of the distances among the network nodes.

**Figure 2:**
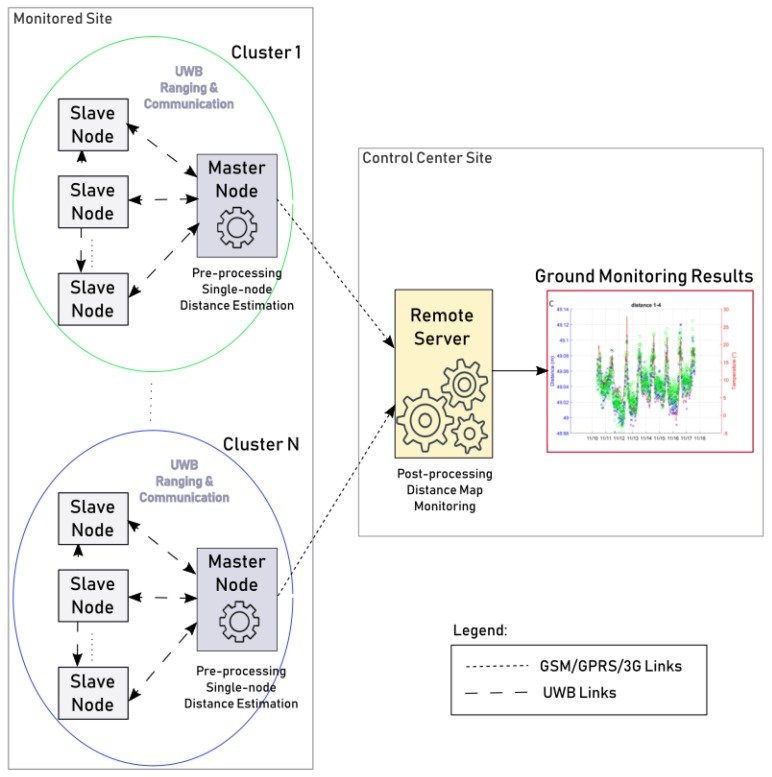
Wi-GIM system architecture.

A high level design of the Wi-GIM system architecture is shown in Figure 2.

In detail, each master node periodically estimates the distances of the slave nodes belonging to its cluster through the adoption of the UWB technology and a pre-processing of the received signals. The obtained information is then transmitted to the remote server via a 3G link for a complete post-processing of all the data coming from the different clusters distributed over the monitored area. As a result, the variation of the distances among the network nodes over time is computed to evaluate the ground movements.

The innovative feature of the proposed system is represented by the exploitation of the impulsive radio frequency technology for achieving an accurate inter-nodes distance estimation, rather than only connectivity among the sensors nodes. This concept is better described in Section 2.2, where both the advantages and drawbacks of the adoption of the UWB technology are highlighted.

Each Wi-GIM network node consists of an electronic board with a micro-controller ARM Cortex M3, a battery, a UWB module and different additional modules, that characterize the specific node function within the network architecture. Mainly, two types of nodes are defined: the master nodes (MNs) and slave nodes (SNs). The former are additionally equipped with a SD memory card, a GPS and GSM/GPRS/3G communication modules, while the latter are optionally equipped with GPS. In Table 1, a list of the network nodes components/modules and their functions is provided.

### 2.2. UWB Technology for Distance Estimation

The UWB technology is extremely efficient for the monitoring of not only fast moving landslide, but also moderate to slow moving ones, thanks to its main characteristics, such as low energy power consumption, high-bandwidth communications over a large portion of the radio spectrum (>500 MHz) and immunity to electromagnetic interference generated by element present on the soil.

**Figure 3:**
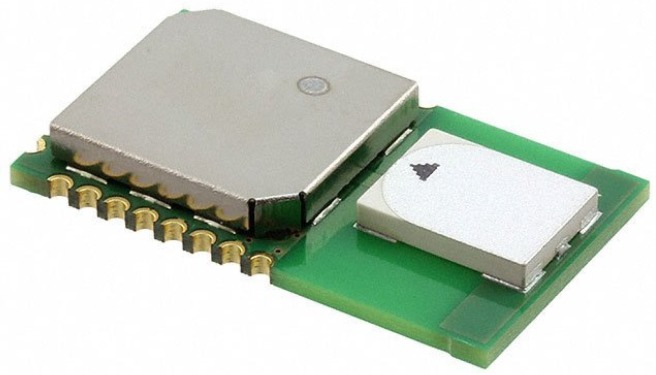
Ultra wideband (UWB) module: Decawave Sensor DWM1000.

**Figure 4:**
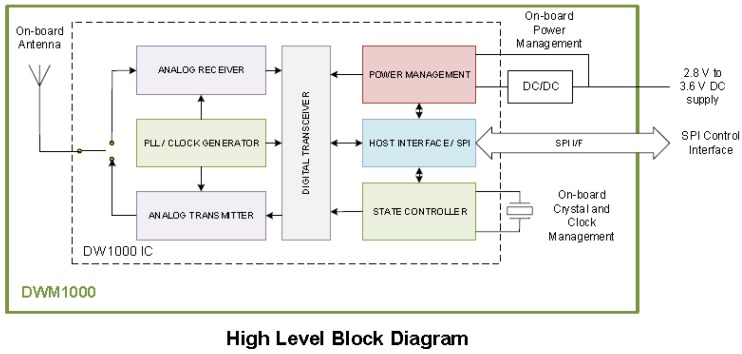
Decawave Sensor DWM1000: Block Diagram.

The impulse-based UWB enables an accurate ranging and high-precision localization of the network nodes [21,22]. The distance estimation is based on the time of flight (TOF) measurement of a radio impulse sent from one UWB module to another. Since the radio impulses travel at the speed of light, the distance between the two modules can be easily estimated, by converting the TOF into a distance. The accuracy of the estimation of the distance is due to the exploitation of the wide band of the transmitted signal, which translates on small impulses over time. In particular, the chosen UWB module, Decawave (Dublin, Ireland) Sensor DWM1000 Module (2017a) (Figure 3 and Figure 4) uses signals with bandwidth of 500 MHz resulting in 0.16 ns-wide pulses. The fine timing resolution of the transmitted impulsive signal drastically limits the signal overlapping at the receiver, allowing an accurate ranging even in places characterized by many reflectors, as in the case of landslides.

The Decawave Module integrates: the Abracon (Spicewood, TX, USA) ACA-107-T dielectric chip antenna (3200–7200 MHz frequency range), all radio frequency circuitry, power management and clock circuitry. It can be used in two-way ranging or TOF location systems to locate assets to a precision of 10 cm and it supports data rates of up to 6.8 Mbps.

The obtained UWB ranging accuracy is detailed in Section 4. Laboratory and field tests show that the UWB ranging systems works fine for node-distances ranging from 60 to 110 m. The performed precision for an inter-nodes distance of 60 m, in line of sight (LOS) conditions, is between 7 and 10 cm.

It is worth highlighting that, in the Wi-GIM system architecture, the UWB chipset provides both localization information of the nodes (i.e., measurement of the inter-node distances) and communication links, avoiding the need of implementing dedicated sensors and therefore representing a promising solution in terms of cost and energy saving.

#### 2.2.1. UWB Two-Way Ranging Technique

Two primary methods can be considered for estimating the distance between a pair of nodes: the first one, based on a single communication between the nodes, expects a strictly fine time synchronization; however, the second one exploits several exchanges of information between the nodes, but does not need any time synchronization [23]. The latter solution has been chosen to be implemented in this system; in particular, an advanced two-way ranging method called symmetric double-sided two-way ranging (SDS-TWR) has been used.

**Figure 5:**
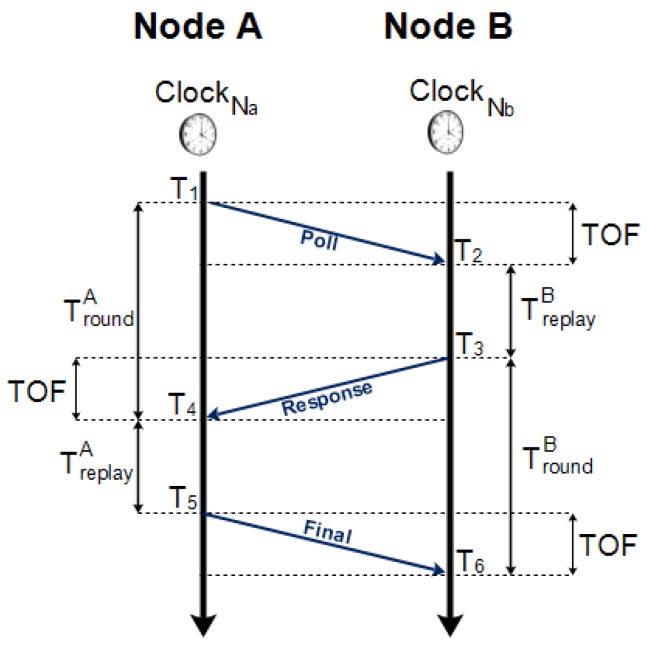
Symmetric double-sided two-way ranging protocol.

Figure 5 illustrates the SDS-TWR protocol, which exploits three communication messages (poll, response and final) between the NodeA and NodeB to determine the TOF. If we consider $T_{round}^{A}=T_{4}-T_{1}$ and $T_{replay}^{A}=T_{5}-T_{4}$, respectively, the round trip time and the reply time which refer to the NodeA clock, $T_{round}^{B}=T_{6}-T_{3}$ and $T_{replay}^{B}=T_{3}-T_{2}$ the round trip time and the reply time which refer to the NodeB clock, then, the TOF between the NodeA and NodeB can be expressed as follows:

(1) $${TOF}_{SDS}=\frac{\left(T_{round}^{A}-T_{replay}^{A}\right)+\left(T_{round}^{B}-T_{replay}^{B}\right)}{4}.$$

The multiplication between the ${TOF}_{SDS}$ and the speed of light in vacuum ($2.99792458\times 10^{8}$) provides the ranging estimation.

If we assume a fixed frequency drifts $e_{A}$ and $e_{B}$ which refer to the NodeA and NodeB clocks, respectively, (1) becomes

(2) $${\hat{TOF}}_{SDS}=\frac{\left(T_{round}^{A}-T_{replay}^{A}\right)\left(1+e_{A}\right)+\left(T_{round}^{B}-T_{replay}^{B}\right)\left(1+e_{B}\right)}{4},$$

and the error due to drifts in the TOF computation is

(3) $${\hat{TOF}}_{SDS}-{TOF}_{SDS}=\frac{{TOF}_{SDS}}{2}\left(e_{A}+e_{B}\right)+\frac{T_{replay}^{B}-T_{replay}^{A}}{4}\left(e_{A}-e_{B}\right).$$

If the $TOF_{SDS}$ is much less than the difference between the two reply times, the error due to clock drifts tends to depend only on the second term in (3), i.e., the product between the time difference of the reply times and the difference between the clock drifts.

### 2.3. Wi-GIM Installation Procedure

The objective of this section is to provide details on the Wi-GIM installation procedure. The installation consists of the following steps: (1) the geologist identifies which are the points over the terrain that must be monitored; (2) the geologist plants the metal posts in the ground; (3) the sensor box (containing battery and all the other modules except the UWB one) is located at the base of the post, while the UWB module is located on the bar at 1 m height. Now, the geologist can start the configuration procedure (software), to define which sensor is the MN and which is the SNs-network (cluster) it has to manage (Section 2.4.1). The geologist, once on the ground, defines the penetration depth of the metal bar. The metal bar is composed by several segments that can be added to variate the total height of the bar. In this way, the bar penetration can be adapted to the type of terrain to be monitored. Moreover, an initial sensors’ position map can be performed by the geologist, using a portable GPS to take the position of the sensors while installing them on the landslide. This allows the geologist to create a geodetic map of the sensor network. However, if the geologist is only interested to the relative movements among the nodes, the initial acquisition of the GPS position can be avoided.

In the proposed system, the GPS module is a low-cost module available in the market and although the board of each node can host it, only the MN has the GPS plugged in. It is important to highlight that the GPS is not used to determine the position of the node, but only to let the MN acquire the global time reference. Once the time reference is acquired, the MN sends this information to all the SNs of its cluster to synchronize them. This procedure is required for the correct functioning of the *sleep* algorithm described in Section 2.4.2.

### 2.4. Processing and Communication Protocols

An overview of the overall functioning of the Wi-GIM monitoring system is provided in the following by analysing the defined algorithms implemented by the master nodes for the coordination operations and the data processing techniques for ground movements tracking.

#### 2.4.1. Network Initialization: Clustering Algorithm

After the network node (MNs and SNs) displacement over the monitored area, the clustering algorithm starts. During the sensors’ displacement in the landslide, the geologist aims to provide LOS condition between the MN and the SNs; however, if a SN needs to be placed behind an obstacle for monitoring purposes, so that the MN is in the NLOS condition, another SN shall be put in LOS with that SN and with the MN enabling the MN to still reach the "blocked" SN. In fact, each MN scans the network to gather information of those SNs that are in LOS condition, then asks these SNs to inform it about the SNs in LOS with them (but in NLOS with the MN). This procedure allows the MN to have a map of all the SNs which are in LOS and the ones reachable through 1 hop thanks to SNs relaying mechanisms. Once the MN completes its SNs map, the cluster is defined and those nodes will be considered for the monitoring strategies. Its worth highlighting that, after the initialization phase, the cluster cannot be modified: the introduction of an additional node requires a new scan of the network. On the other hand, the MN continuously monitor the status of each SN of its cluster (e.g., battery level, correct functioning), as better described in the following.

#### 2.4.2. On Site Master Node: Coordination and Pre-Processing

In the Wi-GIM system architecture, an MN is responsible for the coordination of the SNs of its cluster, which consists of a maximum of 16 nodes, to control the number of transmissions among them, while an SN is in charge of measuring the distance between itself and all surrounding nodes (slaves and master alike).

In order to manage the network resources allocation, the MN sends the activation control signal to an SN, enabling it to occupy the channel and perform the ranging operations. The SNs are activated one at a time. Upon reception of the activation signal, the SN sends an UWB impulsive signal to all its neighbour nodes (LOS nodes), which respond. As the SN completes the distance measurements, by calculating the two-way TOF, it sends the results to the MN and then releases the channel. The MN can now send the activation control signal to another SN and the process is repeated until all the SNs are activated.

As reported in Section 2.4.1, an initial network scanning allows the MN to draw a sketch of the network connections between the nodes, identifying also those SNs that are not directly reachable from the MN. The activation of those SNs is performed through a relay mechanism: the MN activation command is relayed by a LOS SN to the NLOS SN. The IDs of the SNs and the number of hops to reach each specific node are stored in the MN memory card. As the complete scan of the nodes is finished, the MN sends the sleep command and the wake-up time to the slaves. All the SNs deactivate all their power-consuming modules and then reactivate them at the programmed time (*sleep algorithm*). The scheduled measurements activation/deactivation allows an efficient management of the nodes’ battery consumption. The time required for completing a single measurement cycle (which starts with the SNs activation and ends with their deactivation, sleep mode) is around 90 s. To reduce the power consumption, saving the nodes batteries, 2–4 data acquisition per day can be carried out. This measurement frequency can be sufficient for an earth flow, due to its slow temporal evolution. However, in case of WSN monitoring of a landslide for a long time, the battery of each node can be changed without the need of moving the corresponding node. The proposed distance measurements system requires a time reference. As previously mentioned, the MN is equipped with a GPS module, whose function is to provide the GPS time reference to be spread among the SNs before starting the ranging measurements. After collecting all distances, the MN sends a report file to the remote server via 3G connection. The periodical report on the status of the cluster/network includes: the distances measured by all SNs (distances between each node and all the others around) and the corresponding time stamp, the battery level information of the SNs and other useful information (e.g., non-responding nodes, temperature, etc).

It is worth highlighting that all the information exchange between the MN and a single SN is completed in 22 ms. Since an MN can coordinate a cluster of a maximum of 16 SNs, all the measurements and the information (battery level, temperature, etc.) collection of all the nodes take 350–400 ms. Moreover, the MN has a limited time (set to 500 ms) to complete the operations.

#### 2.4.3. Remote Post-Processing

The information collected by the remote server and coming from the MNs shall be processed in a synchronized and coordinated way in order to correctly use the data for the ground monitoring (grid creation). The post-processing is performed by the adoption of specific MATLAB (R2016a, MAthWorks, Natick, MA, USA) functions developed for: (i) data filtering; (ii) automatic comparison of the displacement velocities with respect to a pre-defined thresholds and eventually issue an alerts/notifications; and (iii) plot the displacement diagrams of all the possible combinations of nodes, allowing the operators to perform advanced analyses.

The basic processing consists of:

1. outliers identification and removal (outlier filtering);
2. temperature-dependent error correction;
3. averaging over several measures (statistic filtering);
4. constant offset correction.

The outlier filtering consists of the identification and removal of the unreliable measurements, which, in our case of study, are those distances estimated to be 0 m, or distances estimated to be equal or greater than 1000 m. On the other hand, the statistic filtering consists of performing the average (over time) of the obtained measurements of one specific distance. For example, the geologist could decide to run 10–20 measurements per day and then take the average over all the measurements done in one day, assuring the identification of a real movement if several consecutive distances have changed their values. Averaging can also be done over more than one-day measurements, as in the case of a *slow* landslide. Averaging can reduce the noise and enhance the accuracy. However, averaging over too many measures can limit the identification of when a landslide movement occurs. In our case, averaging is done over the measurements of one day.

The effect of the post-processing and, in particular of the filtering, is shown in Section 4.1.1.

## 3. Laboratory and Field Tests: Wi-GIM System Validation

This section describes the procedures adopted to validate the proposed system in a real environment.

The prototype of the WI-GIM system has been designed and implemented satisfying the requirements of high flexibility, rapid installation times and limited investment costs (low ratio of cost/extension of the area monitored). The test campaign of the WSN prototype includes:

1. *Laboratory tests*. These tests aim to prove the nominal performance of the prototype.
2. *Early calibration tests and sensitivity analyses.* These tests aim at highlighting the system dependence to environmental and physical parameters (snow, rain, obstacles) and lead to the introduction of some technical improvements of the prototype (e.g., firmware redesign for battery management optimization—sleep mode, single hopping mode. They have been carried out in Arcetri, Florence (Central Italy) and result in a higher performance system level.
3. *Landslide application tests.* A target site having different geomorphological characteristics has been considered: it is Roncovetro in Italy. It is characterized by a medium-fast moving landslide.

### 3.1. System Sensitivity Analyses (Arcetri)

As testified by the results reported in Section 4, a set of system sensitivity field tests has been carried out. The test site, chosen both for logistic reasons (easy accessibility) and for its characteristics (outdoor setting with heterogeneous vegetation), is represented by an open environment located in Arcetri, Florence (Central Italy). The absence of landslides or ground movements in this site has permitted to identify the precision of the system and the main factors that could affect the system performance. In fact, real movements have been excluded and the sensor nodes were moved manually only if needed. Both the presence of obstacles (LOS and NLOS conditions) and the temperature variations have been tested in order to evaluate their effect on the performed measurements (see Section 4.2).

**Figure 6:**
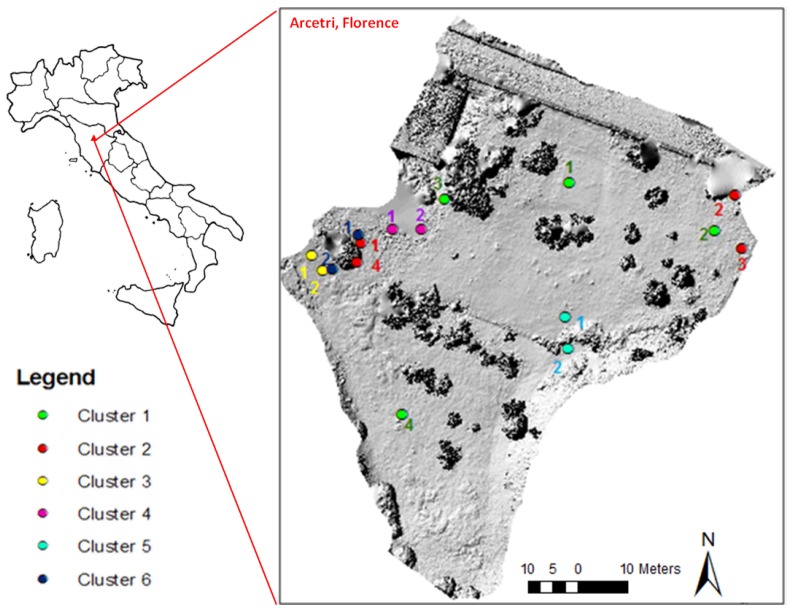
High resolution digital elevation model (DEM) of Arcetri Test site: clusters and node positions. Master nodes (MNs) are indicated with number 1.

Figure 6 shows a high resolution digital elevation model (DEM) of the setting area reconstructed through photogrammetric analysis of aerial images acquired from a drone. This was a very useful tool for the accurate definition of the line of sight between nodes, their relative distance, the presence and characteristics of obstacles for the assessment of their influence on range readings. Six different clusters were deployed for a total of 16 sensors. Table 2 reports the cluster details, including number of nodes, installation and removal dates and the specific scope of the tests they are involved in.

### 3.2. Roncovetro Landslide Monitoring

**Figure 7:**
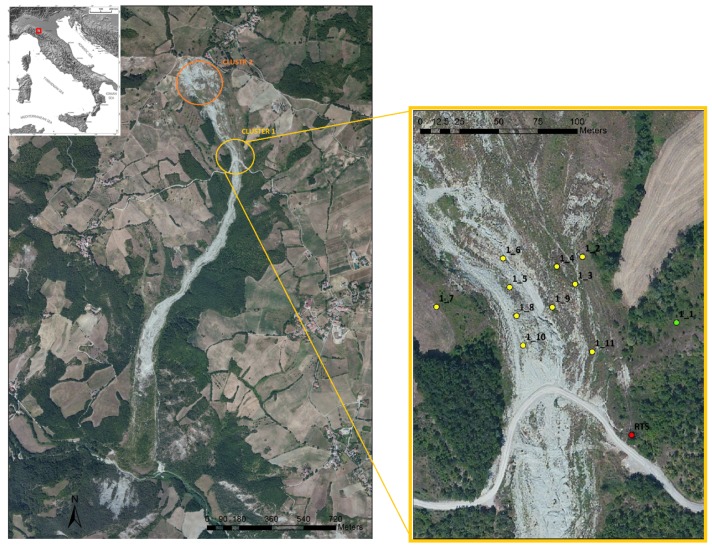
Aerial photograph of the Roncovetro landslide and Wi-GIM clusters. The green dot signifies a sensor which was put outside the landslide, where the terrain is supposed to be not subjected to movements. This point is also used as a reference for mapping.

The Roncovetro landslide is a 2.5 km long complex landslide with a volume of 3 million m${}^{3}$. It is located in the Emilia Romagna Region (Northern Italy) and carves the southern slope of Mount Staffola (where the crown is located) down to the Tassobbio Stream (at the toe), where it partially dams the stream, creating a small seasonal lake (Figure 7). This site has been chosen for test the Wi-GIM system on an actual case of instability [24,25], putting it under stressed conditions. Two different conventional monitoring techniques have been considered for the Roncovetro landslide: terrestrial laser scanning and robotized total station (RTS). Moreover, a weather station has been specifically purchased to evaluate the WI-GIM performance in different meteorological conditions and to identify the main links between the earth-flow activity and the main meteorological parameters.

Two clusters have been installed. Cluster 1 was composed of 11 sensors installed in the middle sector of the Roncovetro earthflow and it was equipped with 1 GPS for time reference and one modem to remotely send acquired data. The time required to deploy, install and configure a cluster in the Roncovetro landslide was less than two hours. The aim of this installation was to evaluate Wi-GIM performance in an active landslide environment with an average inter-nodes distances range of 60–90 m. Three additional nodes were installed in stable areas to back-calculate the position of the others. Cluster 2 was composed of 12 sensors installed in the upper sector of the Roncovetro earthflow and it was equipped with 4 GPS (one for time reference and three for positioning purposes) and one modem to remotely send acquired data. The cluster’s acquisition rate was twice/day.

## 4. Results

This section provides a detailed description of the results achieved during the Wi-GIM experimental test campaign.

In order to validate the proposed system and highlight the benefits and drawbacks coming from the adoption of UWB technology for the landslides monitoring, the WI-GIM performance has been compared with the one obtained by the use of conventional instruments. As a traditional monitoring system, the RTS has been considered. Moreover, the continuous wave radar (CWR) [26] has been tested and considered for comparison in terms of coverage and accuracy.

### 4.1. UWB Distance Estimation and Accuracy

#### 4.1.1. UWB Ranging Measurement Filtering

**Figure 8:**
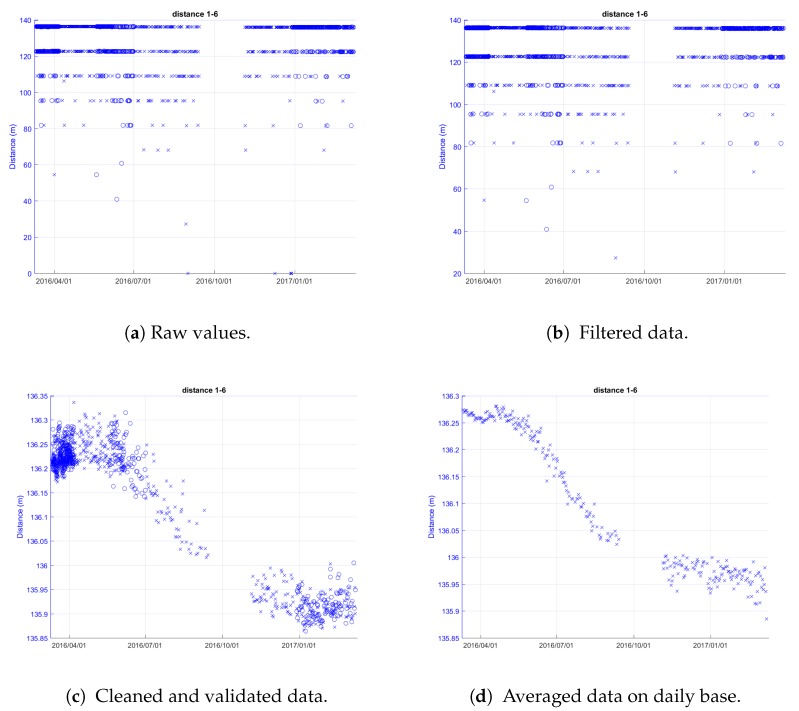
Processing stages on data of distance between nodes 1 and 6 measured by the ultra wideband (UWB) technology over one year (Cluster 1—Roncovetro).

Figure 8 reports an example of the ranging data filtering steps performed by the remote server, starting from the received raw data from the MNs.

In detail, the processing of the ranging measurement of nodes 1–6 (Cluster 1) located in Roncovetro is shown. The distances measured by the MN are plotted as a function of time (raw data) in Figure 8a, where the blue circles and crosses represent the distances measured from one node to the other and vice versa. Where measurement points are extremely dense, they are overlapped. In fact, it is worth highlighting that the system has an intrinsic redundancy, due to the double measurement of the inter-nodes distance: node X measures its distance from node Y and then node Y measures its distance from node X.

The first step of the filtering process consists of the removal of zero and non-numerical values from the raw data (Figure 8b). Then, the outliers (values with a distance greater than 1 m compared to the previous value) are removed (Figure 8c—Outliers Filtering), and finally the mean operation is performed (Figure 8d—Statistic Filtering).

#### 4.1.2. UWB and RTS Ranging Measurement Comparison

**Figure 9:**
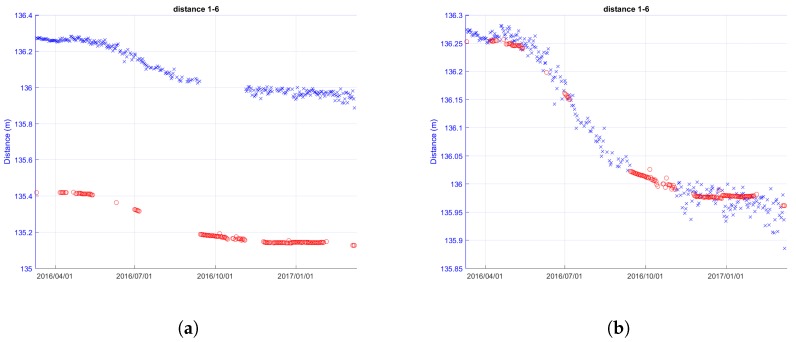
Comparison between the Wi-GIM UWB (blue crosses) and the RTS (red circles) systems to measure the distance between node 1 and 6 (Cluster 1—Roncovetro). (**a**) Data averaged over one day without offset compensation; (**b**) Data averaged over one day with offset compensation.

In Figure 9, the comparison between the UWB and RTS measurements is reported. The former shows an offset relative to the measurement of the absolute distance (Figure 9a). Although the monitoring is not affected by this offset, due to the fact that only the distance variation represents a relevant parameter for early warning, in order to highlight the strong correlation between the UWB and RTS measurements, the offset is suppressed.

#### 4.1.3. UWB and CWR Precision Performance

UWB ranging systems work fine with an inter-nodes distance ranging from 60 m and 110 m. The accuracy obtained working at a distance of 60 m with LOS condition is between 7 cm and 10 cm. The tests have been performed both under laboratory conditions and in the field of application (Table 3).

The UWB module on average has shown an accuracy of 2 cm and a coverage of 110 m in LOS condition and consumes 61 mA/h (when active). The considered UWB chipset is not optimized from an energetic point of view and, in the proposed system, a sleep mode/protocol was specifically designed to overcome the problem of battery duration.

The paper is focused on the use of UWB technology for landslide monitoring; however, a comparison with the CWR is reported for completeness. Reaching sub millimetre accuracy with UWB is a hard challenge. The UWB module uses an impulsive signal, with short-time impulses whose time interval limits the accuracy resolution. The UWB chipset (available in the market in 2014, at the time the project) creates impulse up to 1 ns of time duration. If this limit can be overcome in the future, the UWB ranging accuracy could drop down to sub-millimetre resolution.

CWR uses a radar signal with linear variation of the frequency around a carrier of 24 GHz. The signal hits a (passive) target and an echo comes back. Frequency gap between the transmitted and the echo signals is proportional to the distance. The CWR module has shown an accuracy of 0.7 cm and a coverage of 60 m in LOS condition. A more accurate measure of the distance can be done by evaluating the phase offset between the transmitted signal and echo. However, an ambiguity (2kπ with *k* = 1, 2, 3, …) arises, in this case. A hybrid approach (UWB plus CWR) can be adopted to remove the CWR phase ambiguity and thus improve the performance. The UWB signal is used as reference: the distance is first evaluated by using the UWB technology (accuracy with cm magnitude), the estimated distance is then used to remove the phase ambiguity to the CWR signal, and the distance of the CWR can be thus estimated (accuracy with mm magnitude).

The CWR module consumes a lot of energy. Thus, the radar module could not be used in every node, but only in those close to a power plug.

### 4.2. Analysis of the Influence Factors Effects on Distance Estimation

#### 4.2.1. LOS/NLOS Condition Effect

Although it is important to try to install all the sensors with inter-visibility among them, the presence on an obstacle between the transmitting and the receiving node (NLOS condition) shall be analysed to evaluate the impact on the ranging measurements. Therefore, some tests have been performed to assess the UWB technology sensitivity to obstacles and the corresponding decrease of coverage.

NLOS condition affects the performance of the UWB ranging technique. Since the distance between two nodes is derived by the time-of-flight measure of the UWB impulsive signal from transmitter to receiver, a NLOS condition (e.g., obstacle blocking the direct path) can introduce a bias in the estimation of the distance due to the fact that the first path arriving at the receiver has not run exactly the distance between transmitter and receiver.

The nominal value of the expected error of the distance measurement in LOS condition and one-shot measure is 10 cm. However, as already discussed, the application of processing techniques over several measurements allows for obtaining an error of 2–3 cm, improving the system performance.

The sensor nodes operate effectively within a specified range, which has been experimentally verified: while Decawave data sheet reports 290 m as the maximum value for correct communication and proper ranging operations, the carried out tests has shown a lower value of 150 m in the LOS condition. Both the ranging accuracy and the inter-nodes communication quality can be further reduced in NLOS condition [27]. In this case, the type of obstacle (stone, tree, bush, etc.) impacts on the nodes achieved precision, which has resulted in being in the range of 20–50 cm.

In order to precisely determine the ranging accuracy of the DVM1000, a systematic evaluation approach has been adopted: two nodes were placed at a known distance; then, a set of multiple measurements has been collected and the mean and standard deviation of the measured data have been computed. Some of the experimental results are shown in Table 4.

#### 4.2.2. Temperature Effect

A strong correlation between the measured distances and temperature on a daily scale has been observed during the test campaign.

**Figure 10:**
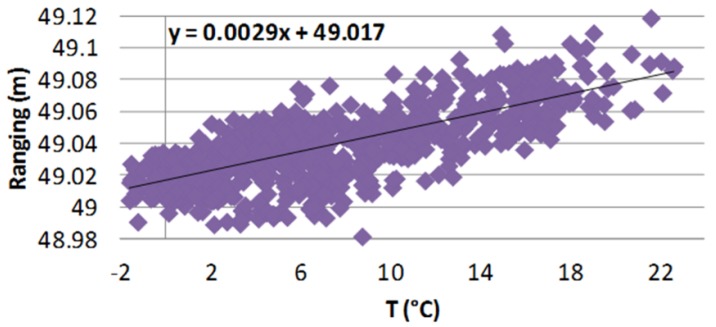
Temperature-measured ranging scatter plot (cluster 1, nodes 1–4, Arcetri test site).

The UWB chipset (as other electronics) has slightly different performance if the temperature where the electronic device is immersed changes (see also Section 5.2). The relation between the environmental temperature and the distance (error) measured by the proposed system is studied: measurements with different temperatures are carried out and then a mathematical (linear) model of this relation is extracted and used to adjust the measure and improve accuracy. The data analysis highlights a linear correlation between these parameters described by a trend line with a slope of 0.0029 (Figure 10).

A correction factor *K* has been computed for each displacement value, considering a standard temperature of 20${}^{\circ }$:

(4) K=(T−20)×m,

where *m* is the trend line slope (Table 5) and *T* is the current temperature.

The difference in the values obtained from the sensors of clusters 1 and 2 is due to the fact that the sensors of cluster 2 were located closer to vegetation compared to the sensors of cluster 1. During day one, the cluster received on average more heat compared to the other; in addition, the humidity created during the night was typically more in cluster 2 than cluster 1.

The application of this correction, consisting of subtracting *K* to every ranging value, results in a consistent reduction of the data variance.

The temperature influence on data has been observed also on a large scale, and in particular on seasonal cycle.

**Figure 11:**
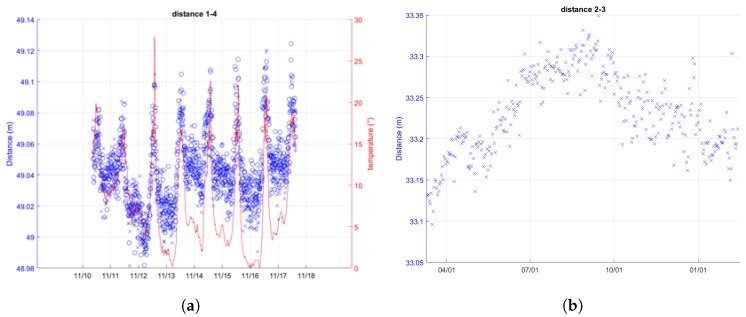
Effect of the temperature on UWB ranging measurements. (**a**) UWB distance measurements between node 1 and 4 (blue) vs. temperature measurements (red). Cluster 1 in Arcetri, from 2016/11/10 to 2016/11/18; (**b**) One-day-averaged UWB ranging measurements between node 2 and 3 over one year (Cluster 1—Roncovetro).

Figure 11 shows the influence of the temperature on the measured distances on a daily scale (Figure 11a) and on seasonal cycle (Figure 11b). Although climatic conditions affect the system performance, the most significant effect can be associated to the temperature variations.

#### 4.2.3. Long Distances Effect

The study of the long distances effect on the overall performance of the Wi-GIM system has been carried out thanks to the Roncovetro experimental test campaign. The inter-nodes distance has been studied in relation with the measurement accuracy: Table 6 reports the standard deviation of measurements for different inter-nodes distances values. In particular, the node pairs 3–4, 1–3 and 1–7 and pairs 2–11, 1–2 and 5–12 are analysed for clusters 1 and 2, respectively.

To evaluate data dispersion, data are analysed during periods of stability for each pair (from 11 March 2016 to 31 May 2016 for cluster 1 and from 1 December 2016 to 6 February 2017 for cluster 2), guaranteeing the measurements independence from actual movements. Moreover, all of the selected pairs were characterized by a percentage of received data higher than 80% and of valid data higher than 60%.

The results of this analysis confirm the absence of any correlation between distance and Wi-GIM precision.

## 5. Discussion

The Wi-GIM system represents a promising solution for a fast characterization of a landslide or an early warning system in case of emergency situations. The system, besides its portability and easy deployment, allows the monitoring of the affected area, providing warning messages also regarding the status (e.g.: battery level) of the cluster nodes and enabling an efficient planning of maintenance operations.

Based on these considerations, in order to highlight the benefits and the limitations of the Wi-GIM system and therefore the UWB technology, in this section, the achieved results are discussed and a comparison among the proposed system and two of the most reliable and precise deployments for landslide monitoring, GB-InSAR and RTS, is reported [28,29]. The choice of GB-InSAR and RTS as a benchmark for our system evaluation is due to their early warning capabilities and easy monitoring of large areas with a relatively small installation effort.

### 5.1. Benefits and Limits of Wi-GIM

The main benefits of the Wi-GIM system for ground deformation monitoring are:

- *Easy and quick installation.* As described in Section 2.3, the Wi-GIM is characterized by an easy and fast deployment and set up. The system set up time is very low: only the MNs are required to be located in specific known positions of a stable area around the unstable and selected to be monitored one, where the SNs are displaced. As an example, a cluster of 10 slave sensors can be installed and configured in less than two hours.
- *Flexibility.* Different kinds of landslide, subsidence cases or other ground deformations can be monitored by the Wi-GIM without requiring specific effort for the system adaptability.
- *Cost effective.* Wi-GIM is characterized by an affordable cost, also making reasonable the monitoring of a large area.
- *Accuracy.* The integrated technologies of Wi-GIM assure the accuracy needed for analyzing different kinds of ground movements. *Obstacle avoidance.* The nodes do not need to be in LOS in order to determine their position.
- *Scalability.* Possibility to monitor movements on a lattice consisting of a large number of nodes.
- *3D monitoring.* Wi-GIM allows the 3D monitoring of the movements, also on a lattice consisting of a large number of nodes;

In Table 7, the benefits of Wi-GIM with respect of RTS and GB-InSAR systems are reported together with the considered evaluation score scale. Wi-GIM outperforms the traditional systems especially in terms of flexibility and deployment. As regards the completeness of information provided by the considered systems, which represents the detectable number of components of the movement vector: GB-InSAR can only provide LOS measurements (1 component), RTS exploits 3D measurements and Wi-GIM can perform from 1D to 3D measurements depending on inter-nodes visibility.

In order to highlight the cost-effectiveness of the proposed system, a cost analysis is carried out for two different cases, involving a monitored area of 500 m${}^{2}$ (case 1) and 100,000 m${}^{2}$ (case 2), respectively. The comparison between WI-GIM and RTS assumes: (i) the optimal installation of Wi-GIM network (all nodes are fully connected and provides 3D vectors) and (ii) RTS prisms for atmospheric noise removal are not considered. In details, five nodes/targets are considered for case 1 and 30 for case 2. However, it is worth noticing that it is not possible to assess an exact proportion between Wi-GIM nodes and RTS prisms, due to their dependence on the installation and quality of data transmission.

Table 8 reports the total cost of the three compared systems. Different costs are included in the total cost: direct, energy consumption, maintenance, installation and data transmission. However, the considerable costs are represented by the direct and installation costs. Due to the too high purchase cost of RTS and GB-InSAR instrumentations, for a possible comparison with the Wi-GIM system, the computed direct costs of RTS and GB-InSAR are represented by the renting cost of the systems for one year. Moreover, it is worth highlighting that the cost reported for the GB-InSAR is low for these kinds of instrumentations.

On the other hand, as shown in Table 9, the main limitations of the proposed system, with respect to RTS GB-InSAR, are represented by the lower durability, precision and maximum range. However, it is worth highlighting that the Wi-GIM is still a prototype system, which may be improved in terms of precision, battery life, node dimensions and cost. The system flexibility and capability to adapt to different operative contexts with very low environmental impact counterbalance these current limitations.

### 5.2. Considerations on the Factors of Influence in the UWB Ranging Accuracy

The accuracy of the UWB ranging measurements can be affected by several factors such as multipath fading and receiver noise [30]. The multipath fading is a feature of the wireless communications and typically occurs when reflectors in the environment surrounding either the transmitter or the receiver create multiple replicas of the transmitted signal which can randomly add up or subtract at the receiver. In UWB communications, contrary to what happens for narrow-band communications, the multipath fading can be drastically limited thanks to the particular structure of the signal [30]. This represents one of the most significant factors for the adoption of UWB technology for landslide monitoring. The receiver noise can be identified as a group of error sources which depend on the quality of the UWB receiver, e.g. the stability of the on-board crystal oscillator. The temperature variation is directly related to the crystal frequency stability. The crystal oscillator on the Decawave DWM1000 module is classified as a room temperature crystal oscillator (RTXO) and its stability may vary within ±30 ppm in a temperature range from $-40^{\circ }$C to $+85^{\circ }$C [31]. As reported in Section 2.2.1, when performing a two-way ranging operation, the frequency drift of the crystal oscillator may cause the increase of the TOF computation error. When the thermal excursion in the environment is high, the distance measurement may vary even within the 24 h of the day, as described in Section 4.2.2. In order to limit the temperature influence in the ranging estimation, a temperature compensated crystal oscillator (TCXO) should be used. Another important factor that can affect the TOF computation is the received signal strength (RSS). Ideally, there is no connection between the RSS and the TOF determination; however, in practice, a bias which depends on the RSS can be observed in the time of arrival determined by the receiving node [32]. This effect can be eliminated with an offset compensation performed in the distance estimation process, as described in Section 4.1.3.

### 5.3. Performance Index

The quality over time of each inter-nodes link has been evaluated by defining the so-called Performance Index (PI), which considers the communication capacity of each couple of nodes and the correctness of the performed measurements. The PI also allows the monitoring of the Wi-GIM performance and its variation over time, enabling an update of the system settings in case of local anomalies detection. It is defined as the product of two parameters: PI=P1×P2:

(5) PI=P1×P2,0≤PI≤1,

where

(6) $$P1=N_{v}/N_{tot}\;,\;0\leq P1\leq 1,$$

(7) P2=1−D0≤P2≤1.

$N_{v}$ is the number of valid distance readings for each couple of nodes (outliers and invalid measurements excluded), and $N_{tot}t$ is the total numbers of readings requested by the MN; *D* is the difference (expressed in meters) between the measured RTS distance and the compensated Wi-GIM distance.

**Figure 12:**
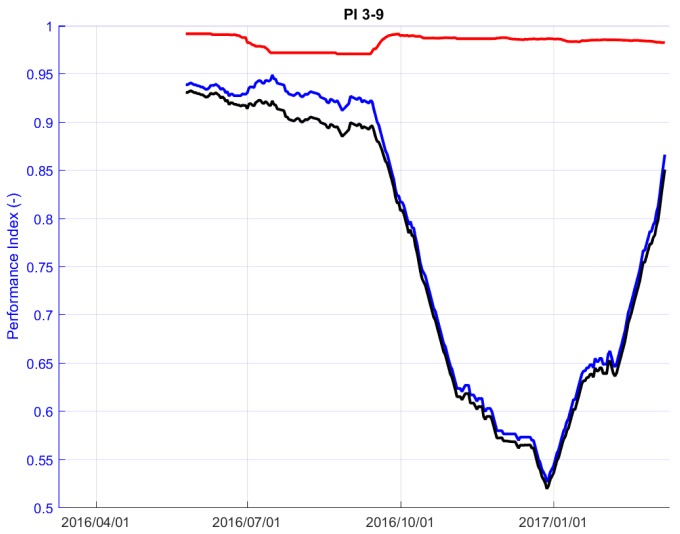
Performance parameters variations over time: *P*1 (blue line), *P*2 (red line) and Performance Index, PI (black line), Roncovetro—cluster 1, node couple: 3–9.

As an example, in Figure 12, the variation of *P*1, *P*2 and PI over time is reported for a couple of nodes of Roncovetro Test site. *P*1 is the parameter that mainly affects PI values; this is mainly due to environmental disturbances (such as vegetation growth, or bad weather conditions) which make the communication more unstable; however, *P*2 shows that the difference between the measurements carried out with RTS and Wi-GIM systems are almost the same, despite the flexibility and cost effectiveness of the UWB technology.

## 6. Conclusions

A new prototypical landslide monitoring instrument based on a low-cost UWB WSN has been developed. The system called Wi-GIM consists of different network nodes organized in clusters (following the master and slave paradigm) and using the UWB to determine their relative distances in order to monitor ground deformations. The system has been validated and tested for landslide early warning in a real context, demonstrating its main promising features for future adoption and highlighting the current limitations that may be improved by further research and industrialization.

The Wi-GIM achieved precision (2–3 cm on filtered data) is lower than the one of the RTS and GB-InSAR systems; this may reduce the range of potential monitoring applications, although it is more than satisfying for many of them. Moreover, the proposed system is still a prototype whose limits may be overcome. Its flexibility, easy deployment and cost-effectiveness characteristics are the main system benefits. Its capability of adaptation to different scenarios together with the the possibility of exploiting additional information, such as the one coming from inertial sensors the slave nodes could be equipped with, leads to the definition of innovative applications, such as the monitoring of fractures and tension cracks (wireless extensometer).

Wi-GIM can be combined with traditional systems for monitoring a selected area, but it can also represent the only monitoring system to be adopted in those areas characterized by resources unavailability. Therefore, it provides the local authorities and Civil Protection with a valuable tool for landslide dynamics analysis and early warning detection.

## Figures and Tables

**Table 1:** Wi-GIM network nodes components and modules.

Component/Module	Function	MN	SN
Micro-controller	ARM Cortex M3 micro-controller.	X	X
Battery	A 12 V, 7.2 Ah lead acid battery for node power supply.	X	X
UWB	Decawave Sensor DWM1000 Module (2017a) for communications and ranging.	X	X
SD memory card	Storage of the IDs of SNs and the number of hops to reach them.	X	
GPS	Time reference signal required for slave coordination.	X	X(*)
GSM/GPRS/3G	Long-range communication modules for data transmission to the remote server.	X	

MN: Master Node; SN: Slave Node; (*) Optional.

**Table 2:** Clusters details of Arcetri Test site.

Cluster No.	Nodes	Installation (yyyy/mm/dd)	Removal (yyyy/mm/dd)	Test Objective
1	1 MN and 3 SNs	2016/10/13	2017/02/06	Inter-visibility between couples of
				nodes of the same network
2	1 MN and 3 SNs	2016/11/11	2017/01/20	Temperature influence on
				the ranging measurement
3	1 MN and 1 SN	2016/12/12	2016/12/27	System behaviour in case
				of obstacles between nodes
4	1 MN and 1 SN	2017/01/03	2017/02/01	System behaviour in case
				of obstacles between nodes
5	1 MN and 1 SN	2017/01/20	2017/02/06	System behaviour in case
				of obstacles between nodes
6	1 MN and 1 SN	2017/02/01	2017/02/16	System behaviour in case
				of obstacles between nodes

**Table 3:** UWB and CWR max distance between nodes and precision.

Technology	Maximum Inter-Node Distance (m)	Precision (m)	Filters	Notes
UWB	110	0.1–0.2	-	LOS
	60	0.07–0.1	Outlier filtering Statistic filtering	Antenna at 1 m to the ground
	60	0.02–0.03	Outlier filtering, statistic filtering,	Antenna at 1 m to the ground
			temperature compensation	
CWR	60	0.07–0.1	Frequency	-
	60	0.01	Frequency + Phase + MUltiple SIgnal	Vertical shift higher than 12 cm
			Classification (MUSIC) Algorithm	needed to discriminate the signal
	10	0.007–0.009	Frequency + Phase	Normal corner reflector

**Table 4:** UWB Ranging Accuracy in LOS/NLOS condition and Indoor/Outdoor operational environment.

Condition	Real Distance (m)	Distance Mean (m)	Standard Dev. (m)	Error (m)
LOS	0.37	0.364	0.022	0.006
Indoor	9.7	9.736	0.020	0.036
	30.32	30.278	0.026	0.042
LOS	2.57	2.623	0.022	0.053
Outdoor	3.37	3.329	0.017	0.041
	25.32	25.391	0.0222	0.071
NLOS	0.39	0.385	0.029	0.005
Indoor	5.42	5.486	0.031	0.066
	29.83	29.789	0.028	0.041

**Table 5:** Example of distances and trend lines slopes of pairs of nodes for clusters 1 and 2 of Arcetri Test Site.

Cluster No.	Nodes	Distance (m)	Trend Line Slope (m)
	1–4	49	0.0029
1	3–4	38	0.0025
	1–3	22	0.0030
	1–4	3	0.0070
2	3–4	67	0.0060
	1–3	67	0.0063

**Table 6:** Standard deviation of 6 pairs of nodes characterized by different inter-node distances (Roncovetro).

Cluster No.	Pair of Nodes	Distance (m)	Standard Deviation (%)
	3–4	15	4.7
1	1–3	70	3.5
	1–7	132	2.6
	2–11	37	3.6
2	1–2	80	3.0
	5–12	130	3.4

**Table 7:** Wi-GIM system benefits analysis compared to RTS and GB-InSAR.

Feature	Wi-GIM	RTS	GB-InSAR
Environmental impact	3	4	4
Installation effort	5	3	4
Influence of rain	4	3	1
Influence of snow	4	3	4
Completeness of information	4	5	5

**Legend:** 1—Poor; 2—Fair; 3—Good; 4—Very good; 5—Excellent.

**Table 8:** Wi-GIM System Cost Analysis.

Case	Monitored Area (m${}^{2}$)	Wi-GIM Cost (€)	RTS Cost (€)	GB-InSAR Cost (€)
1	500	2820	14,750	58,100
2	100,000	5220	18,150	58,100

**Table 9:** Wi-GIM System Limitations with respect to RTS and GB-InSAR.

Feature	Wi-GIM	RTS	GB-InSAR
Durability	2	3	5
Precision	2	4	5
Maximum range	2	5	5

**Legend:** 1—Poor; 2—Fair; 3—Good; 4—Very good; 5—Excellent.
